# Outcomes of newly diagnosed myeloma patients requiring dialysis: renal recovery, importance of rapid response and survival benefit

**DOI:** 10.1038/bcj.2017.49

**Published:** 2017-06-16

**Authors:** M A Dimopoulos, M Roussou, M Gavriatopoulou, D Fotiou, D C Ziogas, M Migkou, I Panagiotidis, E Eleutherakis-Papaiakovou, N Kanellias, E Psimenou, S Marinaki, D Bacharaki, D Mparmparoussi, C Matsouka, S Giannouli, E Terpos, E Kastritis

**Affiliations:** 1 Department of Clinical Therapeutics, School of Medicine, National and Kapodistrian University of Athens, Athens, Greece; 2 Department of Nephrology and Transplantation Unit, Laiko General Hospital, National and Kapodistrian University of Athens, Athens, Greece; 3 Renal Unit, Attiko University Hospital, National and Kapodistrian University of Athens, Athens, Greece; 4 Hematology Division, Alexandra General Hospital, Athens, Greece; 5 Second Department of Internal Medicine, School of Medicine, National and Kapodistrian University of Athens, Athens, Greece

About 50% of newly diagnosed mutilple myeloma (MM) patients (NDMM) have some degree of renal impairment (RI) at presentation, up to 20% have severe acute kidney injury (AKI) and ~1–5% may require extrarenal dialysis, whereas severe RI is associated with high risk of early death and other complications.^1, 2^ Immediate effective anti-myeloma therapy and vigorous supportive care are the cornerstones of management.^2, 3^ However, there are limited data focusing specifically on the management and outcomes of MM patients requiring dialysis as a consequence of MM, mostly including small numbers of patients on dialysis.^4, 5, 6, 7, 8^ Moreover, the use of high cut-off hemodialysis to rapidly reduce the load of nephrotoxic light chains may provide some additional benefit in patients requiring dialysis when treated with bortezomib-based therapies, but the reported results of two randomized studies are controversial.^9, 10^ Thus, we analyzed the outcomes of 52 consecutive NDMM with RI requiring dialysis, which were managed and treated in a single center, to provide data on response to therapy, dialysis discontinuation and survival in unselected patients, outside the context of clinical trials.

The analysis included 52 patients (6.2% of 796 consecutive NDMM) who were treated in the Department of Clinical Therapeutics, and which presented with severe renal failure requiring dialysis, between January 1995 and January 2016. An approval for data collection and publication was obtained by the Alexandra Hospital Scientific Committee. The analysis included all patients who received at least one dose of any therapy. All patients received similar supportive care and dialysis with regular filters. Patients with light-chain amyloidosis, light-chain deposition disease or previously under dialysis due to causes other than myeloma were excluded from the analysis. Renal response was defined as independence from dialysis and discontinuation of this procedure. Myeloma response and progression followed International Myeloma Working Group definitions.^11^ Time to renal recovery (renal response) was calculated from the date of initiation of therapy until the date of the last dialysis session. Survival was calculated from the date of first dose of therapy until the date of last follow-up or death.

The median age of patients requiring dialysis was 69 years (range 37–88), and 68% were >65 years of age; their characteristics are shown in Table 1. High-risk cytogenetics (available in 40 patients) were present in 38%, thus, per revised International Staging System, 75% were revised International Staging System-3 and the rest revised International Staging System-2. Among patients who retained urine flow at presentation, median 24 h Bence Jones proteinuria was 2.2 gr (range 0.1–8.8). Among patients with available Free light chains, median level of involved free-light chain (iFLC) was 9080 mg/l (range 1190–201 000 mg/l) (Table 1). Treatment was bortezomib-based in 43 (82%) patients: 11 (21%) received bortezomib with dexamethasone (VD), 23 (44%) VD plus cyclophosphamide (VCD), 8 (15%) VD plus thalidomide (VTD), 1 (2%) received VD plus doxorubicin (PAD). Nine (17%) patients received non-bortezomib containing regimens: 5 (10%) received thalidomide with high-dose dexamethasone and 4 (7%) high-dose dexamethasone-based regimens alone.

Twenty six (50%) patients achieved a renal response and became dialysis independent. Median time from the first dose of therapy to dialysis independence was 158 days (range 4–336) (Figure 1). Median estimated glomerular filtration rate after dialysis was 45.5 (range 18–84) ml/min/1.73 m^2^.

Age ⩽65 years was associated with higher probability (75 vs 38%) and shorter time to renal response (51 vs 336 days) (*P*=0.027). Among patients older than >75 years, renal recovery rate was 27%. The presence of high-risk cytogenetics (available in 40 patients) or elevated lactate dehydrogenase levels did not impact rates or timing of renal recovery. Hypercalcemia was also not associated with the probability of renal recovery. There was a trend for patients with free-light chain levels ⩾9000 mg/l for lower renal recovery rates and longer time to renal recovery (6 month renal-response rate 41 vs 66%, *P*=0.1, Figure 1).

Among patients treated with bortezomib, dialysis independence rate was 49% (21 out of 43 patients) and triplet bortezomib-based combinations (*n*=32) vs VD alone (N=11) were associated with higher probability of renal responses (57 vs 27% *P*=0.06). High-dose melphalan (HDM) followed by autologous stem cell transplantation was given in five patients while on dialysis. Four of them (80%) became dialysis independent ~1 month after HDM. Median follow-up for all patients is 33 months and median survival is 29 months. Early mortality (within 2 months from start of therapy) was 16%, in most cases because of infectious complications. On intent to treat, 64% achieved ⩾partial response (PR) (CR: 6%, very good partial response: 32%, PR: 26%). Among patients who survived ⩾2 months, ⩾PR was achieved by 76%. At 2 months landmark, patients who achieved at least ⩾PR within the first 2 months had higher rates of dialysis independence (68 vs 27%, *P*=0.004).

Discontinuation of dialysis was associated with a significant improvement in survival: excluding early deaths within the first 2 months, the median overall survival of patients who discontinued dialysis was 63 vs 22 months for patients who remained on dialysis (*P*=0.002). Notably, the survival of patients who discontinued dialysis was similar to that of the rest of MM patients (57 months). Furthermore, the improvement in the survival of those who recovered adequate renal function to discontinue dialysis was independent from myeloma response in landmark analysis, so that median survival (excluding early deaths) was 21 months for patients without myeloma or renal response, 47 months for patients with myeloma response but without renal recovery and 62 months for patients with both myeloma response and renal recovery (*P*=0.047). Importantly, the presence of high-risk cytogenetics by fluorscent *in situ* hybridization (that is, the presence of either del17p, t(4;14) or t(14;16)) was also associated with shorter survival (13 vs 47 months, *P*=0.002). After adjustment for renal recovery, high-risk cytogenetics remained independent predictors of poor outcome in patients requiring dialysis (hazard ratio (HR): 8.76, 95% confidence interval (CI): 1.82-42, *P*=0.007, whereas for renal recovery the HR was 0. 32, 95% CI: 0.08–1, *P*=0.051).

Our current data show that even among patients who present with severe RI requiring dialysis, there is a high probability of about 50% to become dialysis independent without the use of special filters. Furthermore, becoming dialysis independent was associated with a significant survival improvement, approaching the median survival of patients who were not in need of dialysis at the time of diagnosis. Our data for consecutive, unselected patients set the benchmark for the efficacy of new approaches to the management of patients with MM-related renal failure requiring dialysis.

Among patients treated with bortezomib-based regimens, 49% achieved dialysis independence, a high rate of efficacy compared with the historical controls, before the introduction of high cut-off dialyzers.^4, 5, 6, 7, 8, 12^ This rate of efficacy should also be viewed in the context of the recently reported results of the clinical trials of high cut-off hemodialysis. In the EUlite study,^9^ all patients received a bortezomib-based triplet therapy and a high rate of dialysis independence was observed in both the control and high cut-off arm (66 and 58% overall renal recovery rates); these rates are similar to the recovery rates in our patients treated with a bortezomib triplet (57%). In the second prospective study (MYRE),^10^ dialysis independence was achieved in 33 and 43% at 3 months and in 37.5 and 60% at 6 months, in the control and high cut-off arms, respectively. In this study, however, the initial regimen given was a bortezomib doublet (bortezomib plus dexamethasone) followed, after three cycles, with the addition of cyclophosphamide, if adequate hematologic response was not observed. Again, the renal recovery rates in the control arm are similar to those observed in our patients treated with VD (27%). In our analysis, we also observed the importance of early myeloma response: patients who achieved at least PR within the first 2 months had more than double renal recovery rates (68 vs 27%, *P*=0.004). Our data further re-enforce the International Myeloma Working Group recommendation for bortezomib-based triplet as the preferable regimens for patients with MM-related AKI.^1^ Bortezomib may be beneficial in patients with MM-related AKI through two different pathways. First, by reducing rapidly the toxic FLC load as it is a very active anti-plasma cell drug. A second mechanism may implicate the effects of bortezomib in intracellular signaling and gene expression in renal proximal tubular cells, protecting them from apoptotic signaling and pro-fibrotic reaction.^13^

Despite the major impact of severe renal dysfunction requiring dialysis, the presence of high-risk cytogenetics was associated with very poor outcome (median survival of just 13 months), independently of renal function recovery. Importantly, high-risk cytogenetics were present in 38% of patients requiring dialysis, a figure quite high compared to the bulk of myeloma patients, in which high-risk cytogenetics are present in 20–25%.^14, 15^

In conclusion, in patients presenting with acute renal failure in need for dialysis rapid disease response is associated with a high probability of renal function recovery and independence from dialysis. Bortezomib-based triplets offer high rates of myeloma response and subsequent renal response, and remain the standard of care for these patients. Renal recovery is associated with significantly improved survival but high-risk myeloma features are major determinants of outcome.

## Figures and Tables

**Figure 1:**
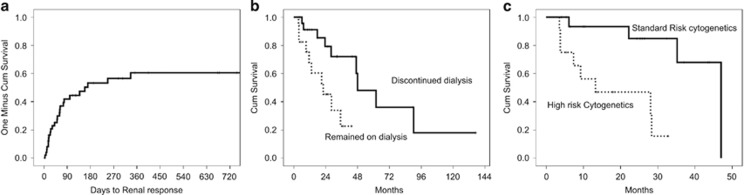
(**a**) time to renal recovery and discontinuation of dialysis in 52 consecutive patients with newly diagnosed myeloma requiring dialysis for end stage renal disease, (**b**) survival of patients who discontinued dialysis at landmark analysis and (**c**) survival according to the presence of high-risk cytogenetics.

**Table 1:** Characteristics of 52 consecutive patients with newly diagnosed myeloma who presented with end stage renal disease requiring dialysis at the time of diagnosis of the disease

	*N=52*
Age (median/range)	69 (37–88)
Age >65	68%
	
Male/female	54/46%
IgG/IgA/IgD/light chain only	30/26/2/42%
Hemoglobin <10 gr/dl	92%
Platelet counts <100 × 10^9^/l	10%
Calcium >11.5 mg/dl	25%
Lactate dehydrogenase >250 IU/l	48%
Serum albumin (median/range) gr/dl	3.5 (2.1–4.6)
B2 microglobulin (median/range) mg/l	21.7 (6–60)
Bence Jone proteinuria gr/24 h (median/range)	2.2 (0.5–8.8)
Involved FLCs mg/l (median/range)	9080 (1190–201 000)
ISS 1/2/3	0/0/100%
High-risk cytogenetics (*N*=40)	38%
Revised International Staging System 1/2/3	0/25/75%
